# Elderly Patients with Mild Cognitive Impairment Exhibit Altered Gut Microbiota Profiles

**DOI:** 10.1155/2021/5578958

**Published:** 2021-11-22

**Authors:** Qiong Pan, Ya-Qian Li, Ke Guo, Min Xue, Yu Gan, Kejian Wang, Da-Bao Xu, Qiu-Yun Tu

**Affiliations:** ^1^Department of Obstetrics and Gynecology, The Third Xiangya Hospital of Central South University, Changsha, China; ^2^Department of Geriatrics, The Third Xiangya Hospital of Central South University, Changsha, China; ^3^Department of Neurology, The Third Xiangya Hospital of Central South University, Changsha, China; ^4^The Third Affiliated Hospital of Shandong First Medical University (Affiliated Hospital of Shandong Academy of Medical Sciences), Jinan, China

## Abstract

**Background:**

As a transitional state between normal aging and Alzheimer's disease (AD), mild cognitive impairment (MCI) is characterized by a worse cognitive decline than that of natural aging. The association between AD and gut microbiota has been reported in a number of studies; however, microbial research regarding MCI remains limited.

**Methods:**

This study examined 48 participants, of whom 22 were MCI cases and 26 were normal control cases. Fecal samples were collected for 16S ribosomal RNA (rRNA) quantitative arrays and bioinformatics analysis.

**Results:**

A principal coordinates analysis (PCoA) and nonmetric multidimensional scaling (NMDS) both demonstrated that the microbial composition of participants with MCI deviated from that of healthy control participants. Multiple bacterial species were significantly increased (e.g., *Staphylococcus intermedius*) or decreased (e.g., *Bacteroides salyersiae*) in samples from the MCI group.

**Conclusion:**

The composition of gut microbiota differed between normal control and MCI cases. This is the first study to identify a signature series of species in the gut microbiota of individuals with MCI. The results provide a new direction for the future development of an early diagnosis and probiotic regimen.

## 1. Background

Mild cognitive impairment (MCI) is regarded as the transitional state between normal aging and Alzheimer's disease (AD) [1]. MCI is a complicated syndrome that is characterized by a cognitive decline greater than that of natural aging, but which does not dramatically interfere with daily life [2]. Epidemiological studies have suggested that the prevalence of MCI is nearly 20% in those older than 65 years old [3]. Despite the seemingly normal status of some MCI patients, several clinical studies have found that most MCI patients will eventually develop AD [4].

Emerging evidence suggests that the disruption of the gut microbiome could undermine mental health. Notably, Zhuang et al.'s clinical research showed that the series of bacteria taxa (e.g., *Bacteroides*, *Ruminococcus*, and Actinobacteria) in AD patients differed from that of control subjects [5]. Vogt et al. identified significant differences in the abundance of Firmicutes (phylum), Bacteroidetes (phylum), and *Bifidobacterium* (genus) in the microbiota of AD cases. Further, correlations have been found between the abundance of certain bacterial genera and biomarkers of AD in cerebrospinal fluid [6]. In AD animal models, the gut microbiome has also been found to be correlated with impaired spatial learning and memory [7]. Li et al. documented similar changes in the gut microbiome among MCI and AD cases [8]. However, there is still very limited evidence concerning the specific abnormalities of gut microbiota in MCI cases compared to those in normal control cases.

In the present study, potential alterations in the gut microbiota of cognitive impairment patients were investigated with 16S ribosomal RNA (rRNA) quantitative microarray, a novel high-throughput biotechnology that quantifies various bacteria taxa without conventional culture-based procedures [9, 10]. We also examined whether the composition of microbiota was correlated to certain mental status parameters of cognitive impairment.

## 2. Methods

### 2.1. Study Design and Sample Collection

MCI (*n* = 22) and control (*n* = 26) participants were recruited from The Third Xiangya Hospital of Central South University. MCI was diagnosed by the Geriatric Department of The Third Xiangya Hospital of Central South University, China. This study was approved by the Ethics Committee of The Third Xiangya Hospital of Central South University and performed in accordance with the relevant guidelines and regulations. Written informed consent was obtained before the study from the participants or guardians on behalf of those participants with impaired cognition. The exclusion criteria for the study were as follows: use of antibiotics within the last 6 months, large doses of probiotics consumed in the last 3 months, current gastrointestinal disorders (e.g., chronic diarrhea, inflammatory bowel disease, or infectious gastroenteritis), and/or major gastrointestinal surgery in the past 3 years. Fresh stool samples were collected and saved in sampling tubes with preservative solution. The tubes and preservative solution were provided by Halgen Ltd. (Zhongshan, China).

### 2.2. DNA Extraction and Labeling

Bacterial DNA was extracted from the stool samples using the Stool DNA Extraction Kit (Halgen Ltd.) in accordance with the procedures described in the product instruction manual. Following previously published protocols [11], a universal primer pair was used to amplify the DNA in the V1-V9 regions of the 16S rRNA gene. Agarose gel electrophoresis was run to check the success of the PCR amplification.

### 2.3. Microarray Hybridization

Here, again, previous protocols [11] were followed to carry out the hybridization between samples and probes on the microarray (3.5 h at 37°C). Immediately after hybridization, the microarray was washed in 2x SSC, 0.25% Triton X-100, 0.25% sodium dodecyl sulfate (SDS), and 1x Dye Protector (Halgen Ltd.) for 15 mins at 63°C and then rinsed in 1x Dye Protector. Finally, a dual-channel scanner was used to quantify the intensity of hybridization.

### 2.4. Data Analysis

Alpha diversity was calculated using QIIME software [12] (and its default parameters). The differences of the alpha diversities between the groups were calculated using a Wilcoxon rank-sum test. A principal coordinates analysis (PCoA) and nonmetric multidimensional scaling (NMDS) were performed by QIIME modules and visualized by R package (version 3.5.2). To detect any statistical differences in the beta diversity metrics between the groups, a permutational multivariate analysis of variance (PERMANOVA) was used in the vegan package in R. A linear discriminant effect size (LEfSe) [13] analysis was performed to analyze any differences in the bacterial species between the groups. The *P* value for each species was calculated using a Kruskal-Wallis test and Wilcoxon test. Unsupervised random forest clustering and receiver operating characteristic curve (ROC curve) proportional hazards statistics were also determined using R. Cross-validations were performed by a leave-one-out test in random forest clustering to reduce the effect of overfitting.

## 3. Results

### 3.1. Demographic Data of Study Participants

A total of 48 participants (comprising 22 MCI cases and 26 control cases) were recruited from The Third Xiangya Hospital of Central South University. The gut microbiota of fecal samples collected by clinicians were analyzed (see Methods). As Table 1 shows, the MCI and control groups did not differ with respect to the female-to-male ratio, body mass index (BMI), education, major preexisting conditions, or physiological variables, and only a minor difference in average age was observed (*P* = 0.046). However, there were significant differences (*P* < 0.01) between the two groups in terms of mental state and cognitive function as measured by Mini-Mental State Examination scores and the Index for Activities of Daily Living.

### 3.2. MCI Cases Harbored an Altered Gut Microbiota

**Figure 1:**
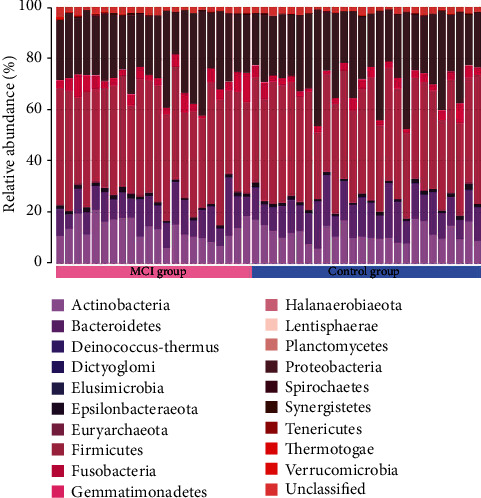
Gut microbiota composition. Proportion of the different phyla (represented by different colors) detected in the two groups.

Compositional analysis revealed the presence of 19 phyla across all samples; however, only 7 of the 19 phyla were above the mean value of 1% of the total abundance (see Figure 1(a)). The relative abundance of *Bacteroidetes* was found to be lower in MCI cases than in control cases. Conversely, *Fusobacteria* were significantly more abundant in MCI cases than in control cases.

**Figure 2:**
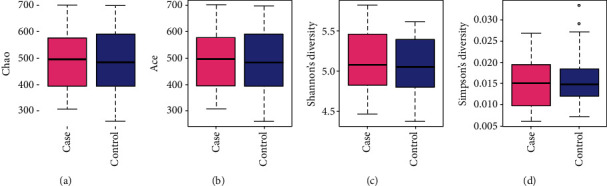
Alpha diversity in the MCI and control groups as represented by Chao index (a), ACE index (b), Shannon diversity (c), and Simpson diversity (d).

**Figure 3:**
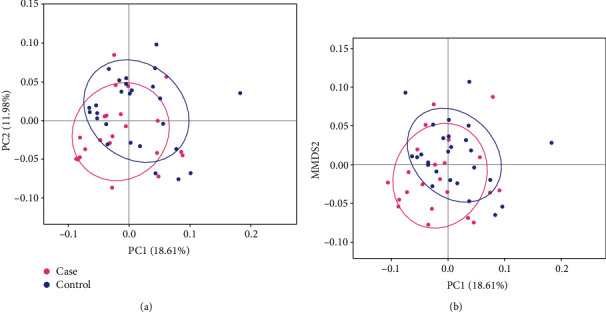
Analysis of beta diversity based on PCoA (a) and NMDS (b) with weighted UniFrac distance. MCI cases and control subjects are denoted with pink and blue nodes, respectively.

The analysis of alpha diversity (see Figure 2) included the calculation of Chao, ACE, Shannon, and Simpson indices; however, no significant difference between the MCI and control groups was detected (*P* > 0.05). The analysis of beta diversity, including the PCoA and NMDS, demonstrated that the gut microbiota profiles of the MCI cases clustered apart from those of control subjects (see Figure 3; PERMANOVA *P* = 0.048). Such separation indicated that MCI-related changes may occur in certain bacterial taxa.

### 3.3. Association between Bacterial Abundance and Cognitive Status

**Figure 4:**
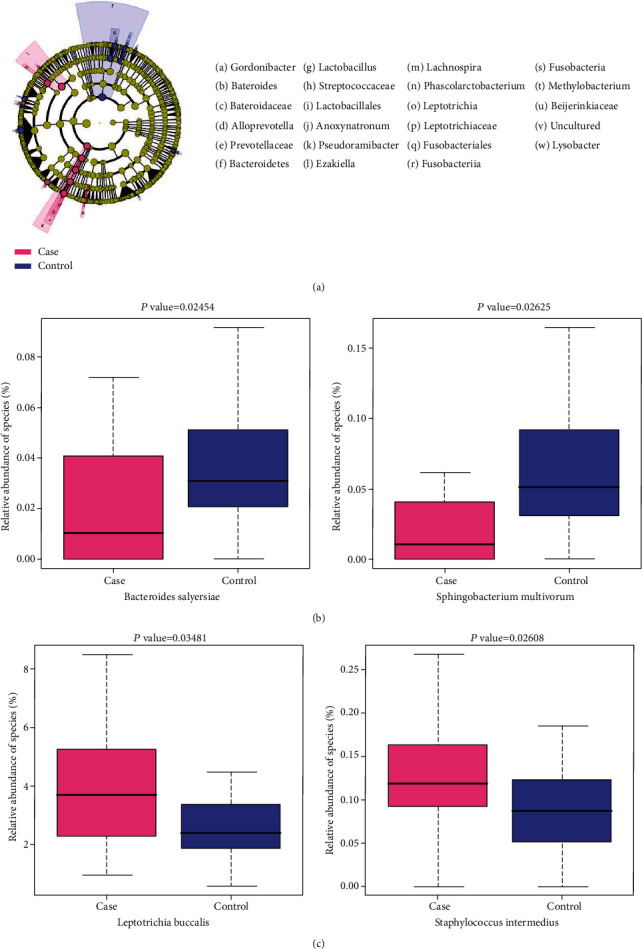
Bacterial taxa differentially represented in samples from MCI cases compared to control cases. (a) Cladograms generated by LEfSe software show the differences. Nodes in pink and blue indicate taxa that were enriched in the MCI and control groups, respectively. (b) Representative species with decreased abundance in the MCI group. (c) Representative species with increased abundance in the MCI group.

Given the MCI-related alterations in gut microbiota, an in-depth analysis was undertaken using the LEfSe analysis (see Methods). A series of bacterial taxa were identified as displaying a differential abundance between the MCI cases and normal controls (see Figure 4(a) and Table S1). The 16S rRNA microarray revealed the significant enrichment of 9 species (e.g., *Staphylococcus intermedius*, Figure 4(b)) and the attenuation of 25 species (e.g., *Bacteroides salyersiae*; see Figure 4(c)) among the MCI cases, particularly at the species level.

## 4. Discussion

MCI has important implications for the health of the elderly, as individuals with a history of MCI are more likely to develop AD in the long term [14, 15]. A number of studies have provided compelling evidence that dysbiosis plays an important role in the pathogenesis of AD [16] and MCI [17]. However, to date, there remains a lack of extensive research on the correlation between gut microbiome and MCI. In the present study, we examined gut dysbiosis in MCI cases. We found that the relationship between bacterial taxonomic profiles and MCI was not characterized by altered alpha diversity. However, a beta diversity analysis visualized a distinction between MCI and control groups that suggested an abnormal depletion of certain bacterial taxa in the MCI cases. Notably, the reduction of *Bacteroides salyersiae* and *Bacteroides gallinarum* in MCI cases was in line with previous research on AD. Zhuang et al. reported the depletion of the *Bacteroides* in AD cases but did not specify the depleted species by conventional 16S rRNA sequencing [5]. *Bacteroides fragilis*, another species of *Bacteroides*, has also been reported to be decreased in patients with cognitive impairment and brain amyloidosis [18]. Our findings further corroborate the relevance of the *Bacteroides* genus in the gut microbiota in relation to neurodegenerative diseases and identified two more species that can be used as potential biomarkers in the early detection of MCI or AD [19].

Conversely, the enrichment of certain taxa in MCI cases was also found to be related to neurodegeneration. For example, *Staphylococcus intermedius* and *Staphylococcus lentus* of the *Staphylococcus* genus were significantly more abundant in the MCI group than in the control group. A number of studies have suggested that *Staphylococcus* is involved in the generation of extracellular amyloid fibers [20] through multiple mechanisms, including the regulation of phenol soluble modulins (PSMs). The PSMs produced by *Staphylococcus* have been documented to form amyloid fibers in biofilms [21]. To date, most published research has noted the relevance of *Staphylococcus aureus*; however, our results identified two other species of the same genus, thus expanding the scope for investigating the role played by *Staphylococcus* in neurodegeneration.

Our study is notable, as it has certain technical advantages. First, unlike other studies on AD, the present research initiatively investigated MCI as a separate phenotype, thus providing unique insights into the progression of MCI to AD. Second, unlike 16S rRNA sequencing that only provides genus-level data [22], the 16S quantitative microarray technology enabled us to scrutinize MCI-related alterations in the gut microbiome at the species level. In addition to shedding light on the role played by the brain-gut axis in the process of neurodegeneration, our findings might also promote the development of more precise diagnostic methods for MCI that are based on gut microbiota signatures.

However, a few limitations of our study should also be taken into consideration. First, due to the complex process of participant enrollment and the application of our strict exclusion criteria, the size of the sample was restricted. Due to the relatively low sample size, the occurrence of beta error cannot be completely excluded, particularly as statistical significance was not reached. This might explain the discovery of some differentially abundant taxa in our MCI samples that were not discussed in previously published results on AD patients. Second, since gut microbiota is associated with a variety of diseases, although several gastrointestinal disorders have been considered in patient enrollment, our sample size restricted the ability to adopt more comprehensive exclusion criteria. Third, this study lacks information on diet of individual participants, while recently several studies suggested the important role of diet in shaping gut microbiome. As a result, potential dietary differences may still affect the results to some extent. Fourth, we observed a barely detectable age difference between case and control groups, which may slightly affect the results of statistical analysis. In addition, as all of the participants were recruited from the same hospital, potential regional variations among gut microbiota could not be evaluated. We intend to conduct further multicenter clinical research with a larger sample size to more thoroughly investigate gut microbiota among MCI subjects across different regions.

In conclusion, the present study provided new evidence of abnormalities in the gut microbiota of MCI cases in relation to those of control subjects. Our results can be used to guide the development of a microbiota-based diagnosis in the early detection of MCI and subsequent AD. Additionally, the new-found alterations in the bacteria of MCI cases may provide clues for a probiotic regimen that could alleviate age-associated cognitive decline.

## Figures and Tables

**Table 1:** Baseline characteristics of study subjects.

	MCI case (*n* = 22)	Control (*n* = 26)	*P* value
Age (yrs, mean ± SD)	71.45 ± 8.03	67.31 ± 5.27	0.046
Sex (female/male)	14/8	19/7	0.543
BMI (kg/m^2^, mean ± SD)	23.78 ± 3.98	22.05 ± 5.10	0.194
Education			0.87
Illiteracy	5	7	
Elementary school	11	11	
High school	6	8	
MMSE score (mean ± SD)	15.55 ± 4.50	23.96 ± 2.84	7.81 × 10^−9^
Hamilton Depression Rating Scale (HAM-D, mean ± SD)	5.09 ± 4.85	4.08 ± 3.63	0.424
Index for Activities of Daily Living (ADL, mean ± SD)	25.95 ± 8.14	21.04 ± 1.28	0.01
Major preexisting conditions			
Cerebrovascular diseases	9	9	0.654
Cardiopathy	5	4	0.781
Hypertension	2	5	0.561
Diabetes	3	1	0.485
Respiratory tract diseases	6	5	0.509
Genital diseases	7	10	0.632
Physiological data			
Blood glucose (mmol/L)	5.50	4.82	0.078
TAG (mmol/L)	1.93	1.59	0.362
TC (mmol/L)	4.63	4.59	0.903
HDL-cholesterol (mmol/L)	1.21	1.08	0.379
LDL-cholesterol (mmol/L)	2.25	2.34	0.693

## Data Availability

The datasets used and/or analyzed in the current study are available from the corresponding authors on reasonable request.

## Supplementary Materials

Supplementary Materials Table S1: bacterial species with significant change of relative abundance between the MCI and control groups.
