# Supplementary material for: Elderly Patients with Mild Cognitive Impairment Exhibit Altered Gut Microbiota Profiles
Source: J Immunol Res. 2021 Nov 22;2021:5578958. doi: 10.1155/2021/5578958 (PMC8635943; doi:10.1155/2021/5578958)
Supplement: Supplementary Materials — Table S1: bacterial species with significant change of relative abundance between the MCI and control groups. [file 5578958.f1.pdf]

Table S1. Bacterial species with significant change of relative abundance between MCI and control groups.

Level	Phylum	Genus	Species	Alteration in MCI	p-value
Species	Firmicutes	Staphylococcus	Staphylococcus lentus	Increased	0.00072
Species	Proteobacteria	Sphingomonas	Sphingomonas panni	Increased	0.00137
Species	Firmicutes	Staphylococcus	Staphylococcus intermedius	Increased	0.00691
Species	Bacteroidetes	Hydrobacter	Hydrobacter penzbergensis	Increased	0.01794
Species	Actinobacteria	Bifidobacterium	Bifidobacterium callitrichos	Increased	0.02004
Species	Actinobacteria	Corynebacterium	Corynebacterium matruchotii	Increased	0.02393
Species	Firmicutes	Paenibacillus	Paenibacillus senegalensis	Increased	0.02535
Species	Firmicutes	Clostridium sensu stricto	Clostridium felsineum	Increased	0.02611
Species	Firmicutes	Staphylococcus	Staphylococcus saprophyticus	Increased	0.02654
Species	Firmicutes	Lactobacillus	Bacillus amyloliquefaciens	Increased	0.0276
Species	Verrucomicrobia	Akkermansia	Akkermansia glycaniphila	Increased	0.02846
Species	Actinobacteria	Brevibacterium	Brevibacterium linens	Increased	0.02846
Species	Bacteroidetes	Hymenobacter	Hymenobacter rigui	Increased	0.0293
Species	Fusobacteria	Leptotrichia	Leptotrichia buccalis	Increased	0.0298
Species	Firmicutes	Pseudoramibacter	Pseudoramibacter alactolyticus	Increased	0.03269
Species	Actinobacteria	Corynebacterium 1	Corynebacterium ureicelerivorans	Increased	0.03341
Species	Firmicutes	Streptococcus	Streptococcus equi	Increased	0.03441
Species	Proteobacteria	Eikenella	Eikenella corrodens	Increased	0.03614
Species	Synergistetes	Pyramidobacter	Pyramidobacter piscolens	Increased	0.03805
Species	Bacteroidetes	Alistipes	Alistipes indistinctus	Increased	0.04025
Species	Epsilonbacteraeota	Helicobacter	Helicobacter pametensis	Increased	0.04051
Species	Fusobacteria	Fusobacterium	Fusobacterium necrogenes	Increased	0.04189
Species	Actinobacteria	Bifidobacterium	Bifidobacterium longum	Increased	0.04257
Species	Firmicutes	Anaerostipes	Anaerostipes caccae	Increased	0.04565
Species	Firmicutes	Johnsonella	Johnsonella ignava	Increased	0.04797
Species	Bacteroidetes	Parabacteroides	Parabacteroides johnsonii	Increased	0.04812
Species	Firmicutes	Megamonas	Megamonas rupellensis	Increased	0.04889
Species	Actinobacteria	Corynebacterium	Corynebacterium ulcerans	Increased	0.0493
Species	Firmicutes	Lactobacillus	Lactobacillus alimentarius	Increased	0.04933
Species	Firmicutes	Leuconostoc	Pediococcus pentosaceus	Decreased	0.00039
Species	Proteobacteria	Pseudomonas	Pseudomonas monteilii	Decreased	0.00115
Species	Firmicutes	Lachnospira	Lactobacillus rogosae	Decreased	0.00137
Species	Firmicutes	Bacillus	Cloacibacterium normanense	Decreased	0.0033
Species	Firmicutes	Ezakiella	Bacteroides coagulans	Decreased	0.00503
Species	Bacteroidetes	Porphyromonas	Porphyromonas gingivalis	Decreased	0.00538
Species	Firmicutes	Lachnospira	Lachnospira pectinoschiza	Decreased	0.0076
Species	Bacteroidetes	Sphingobacterium	Sphingobacterium multivorum	Decreased	0.00815
Species	Firmicutes	Roseburia	Roseburia intestinalis	Decreased	0.00876
Species	Proteobacteria	uncultured	Achromobacter denitrificans	Decreased	0.01029
Species	Bacteroidetes	Alloprevotella	Alloprevotella tannerae	Decreased	0.01029

Species	Fusobacteria	Fusobacterium	Fusobacterium naviforme	Decreased	0.01124
Species	Firmicutes	Anoxynatronum	Anoxynatronum sibiricum	Decreased	0.01229
Species	Firmicutes	Clostridium sensu stricto 11	Clostridium collagenovorans	Decreased	0.01394
Species	Firmicutes	Clostridium sensu stricto 1	Clostridium chartatabidum	Decreased	0.01403
Species	Firmicutes	Lachnoanaerobaculum	Lachnoanaerobaculum saburreum	Decreased	0.01831
Species	Actinobacteria	Gordonibacter	Gordonibacter pamelaeae	Decreased	0.0193
Species	Actinobacteria	Kocuria	Kocuria halotolerans	Decreased	0.02052
Species	Bacteroidetes	Bacteroides	Bacteroides salyersiae	Decreased	0.0208
Species	Firmicutes	Dialister	Bacteroides xylanisolvens	Decreased	0.02654
Species	Proteobacteria	Aquabacterium	Aquabacterium commune	Decreased	0.02855
Species	Actinobacteria	Microbacterium	Microbacterium gubbeenense	Decreased	0.03662
Species	Firmicutes	Streptococcus	Streptococcus viridans	Decreased	0.03663
Species	Proteobacteria	Vibrio	Pseudomonas fluorescens	Decreased	0.03755
Species	Proteobacteria	Pantoea	Enterobacter cancerogenus	Decreased	0.03756
Species	Proteobacteria	Escherichia	Escherichia hermannii	Decreased	0.04051
